# Identification and Evaluation of Reliable Reference Genes for Quantitative Real-Time PCR Analysis in Tea Plant (*Camellia sinensis* (L.) O. Kuntze)

**DOI:** 10.3390/ijms151222155

**Published:** 2014-12-02

**Authors:** Xinyuan Hao, David P. Horvath, Wun S. Chao, Yajun Yang, Xinchao Wang, Bin Xiao

**Affiliations:** 1 College of Horticulture, Northwest A&F University, Yangling 712100, Shaanxi, China; E-Mail: amery.hao@yahoo.com; 2 USDA-Agricultural Research Service, Biosciences Research Lab, Sunflower and Plant Biology Research Unit, 1605 Albrecht Blvd N, Fargo, ND 58102, USA; E-Mails: david.horvath@ars.usda.gov (D.P.H.); wun.chao@ars.usda.gov (W.S.C.); 3 Tea Research Institute, Chinese Academy of Agricultural Sciences, No. 9 South Meiling Road, Hangzhou 310008, Zhejiang, China; E-Mail: xcw75@tricaas.com; 4 National Center for Tea Improvement, Key Laboratory of Tea Biology and Resources Utilization, Ministry of Agriculture, No. 9 South Meiling Road, Hangzhou 310008, Zhejiang, China

**Keywords:** *Camellia sinensis*, reference gene, qRT-PCR, tea plant, gene expression, normalization

## Abstract

Reliable reference selection for the accurate quantification of gene expression under various experimental conditions is a crucial step in qRT-PCR normalization. To date, only a few housekeeping genes have been identified and used as reference genes in tea plant. The validity of those reference genes are not clear since their expression stabilities have not been rigorously examined. To identify more appropriate reference genes for qRT-PCR studies on tea plant, we examined the expression stability of 11 candidate reference genes from three different sources: the orthologs of *Arabidopsis* traditional reference genes and stably expressed genes identified from whole-genome GeneChip studies, together with three housekeeping gene commonly used in tea plant research. We evaluated the transcript levels of these genes in 94 experimental samples. The expression stabilities of these 11 genes were ranked using four different computation programs including geNorm, Normfinder, BestKeeper, and the comparative ∆C_T_ method. Results showed that the three commonly used housekeeping genes of *CsTUBULIN1*, *CsACINT1* and *Cs18S rRNA1* together with *CsUBQ1* were the most unstable genes in all sample ranking order. However, *CsPTB1*, *CsEF1*, *CsSAND1*, *CsCLATHRIN1* and *CsUBC1* were the top five appropriate reference genes for qRT-PCR analysis in complex experimental conditions.

## 1. Introduction

Gene expression analysis is an important approach, providing insight into the genetic and developmental mechanisms in biological research. Quantitative real-time polymerase chain reaction (qRT-PCR) has become a critical tool for rapid and reliable quantification of gene transcript levels due to its simplicity, specificity, and sensitivity [1,2,3]. However, a successful qRT-PCR assay relies on many factors, including the integrity of purified RNA, reverse transcription efficiency, primer design, detection chemistry selection, and data analysis [4,5]. Because experimental variation is unavoidable, an accurate method of normalization is essential in qRT-PCR assays [6]. Normalizing results with a stably-expressed internal reference gene is a simple and popular method for controlling error in qRT-PCR, and is generally preferred over other normalization strategies based on sample size, total RNA quantification, and spiked control molecules [7]. Therefore, the selection and validation of endogenous reference genes for qRT-PCR in each species is necessary.

Often actin, ubiquitin, or 18S rRNA, or other housekeeping genes are used without any evidence that such genes are stably expressed across the experimental conditions described. These genes were reasonable choices for hybridization-based expression analyses where the probes for these housekeeping genes often hybridized to multiple gene family members and thus were representative of the overall gene expression. However, we now know from RNAseq studies that individual gene family members can have very different expression values in regards to both tissue specificity and response to environmental changes. Because qRT-PCR is often gene specific, it is no longer acceptable to arbitrarily choose a common housekeeping gene as an internal control unless there is evidence that it is likely to show stable expression across the samples of any given experiment. Indeed, several published results examining the suitability of various commonly used control genes indicate substantial variation in expression of commonly used control genes, but have identified several genes that appear to be more appropriate for use as controls for qRT-PCR [8,9]. However, an examination of such results suggests that the best of control genes can vary between species and even between experimental conditions. Thus, an evidence-based choice of control genes is needed for any given gene expression analysis using qRT-PCR.

Tea is the most consumed beverage in the world aside from water [10], and has many beneficial health effects [11,12,13]. Tea plant (*Camellia sinensis*), is an important cash crop, and is grown commercially in about 30 different countries. To improve the quality and productivity of tea plant, an increasing number of researchers are investigating the physiology, biochemistry and metabolism of tea plant using molecular biotechnology [14]. Singh *et al.* [15] evaluated 26S rRNA genes by non-quantitative RT-PCR in tea plant together with six other plant species. Gohain *et al.* [16] validated the suitability of primers that amplify 18S rRNAs, 26s rRNAs, ribulose-1,5-bisphosphates (RuBPs) and actins as potential reference genes, but only examined transcript levels in tea leaves. To date, no comprehensive study on endogenous reference genes for qRT-PCR analysis has been done in tea plant. Surprisingly, various members of the housekeeping gene families encoding actins, tubulins, glyceraldehyde 3-phosphate dehydrogenases (GAPDHs), 18S rRNAs and 26S rRNAs are the only reference genes currently used in gene expression analysis of tea plant [17,18,19,20,21,22,23,24]. Due to the increased sensitivity and dynamic range of real-time PCR over traditional quantization techniques, an increasing number of studies have documented the poor stability of commonly known housekeeping genes like β-actins, GAPDHs and 18S rRNAs [25,26]. To avoid erroneous results, the expression stability of chosen reference gene needs to be validated depending on individual samples/experiments rather than relying on previously published materials unless validation of the chosen reference genes was demonstrated [6]. Recently, studies on response of tea plant to cold, seasonal dormancy, and tissue specific gene expression have become subjects of interest [17,19,22,23]. Thus, the validation of highly stable reference genes under these conditions is imperative.

To identify suitable reference genes for normalizing qRT-PCR tea plant gene expression analysis specifically suitable for the expression analysis in several different experimental conditions, we examined the expression stability of 11 candidate reference genes from three different sources: the orthologs of traditional *Arabidopsis* reference gene family members encoding GAPDHs, elongation factors (EFs), ubiquitins (UBQs) and ubiquitin carrier proteins (UBCs), stably expressed genes identified from whole-genome GeneChip studies that encode clathrins, MONENSIN SENSITIVITY1 (MON1 or SAND), tonoplast intrinsic proteins (TIPs) and polypyrimidine tract-binding proteins (PTBs) [27], and three housekeeping gene currently used in tea plant research that encode 18S rRNAs, actins and tubulins. We evaluated the transcript levels of these genes together with two tea plant target genes *SHORT VEGETATIVE PHASE 1* (*CsSVP1*) and *FLOWERING LOCUS T 1* (*CsFT1*) in 94 experimental samples consisting of diurnal variety (24 samples), different organs (18 samples), different duration of cold stress in leaves and shoots (each contains 18 samples), and different time points after auxin antagonist auxinole treatment (eight samples) and lanolin treatment (eight samples). Accurate quantification of cDNA synthesis products was performed using radioactivity detection of [α-^32^P]-dCTP incorporation. The expression stability of candidate reference genes was ranked using comparative ∆*C*_T_, BestKeeper, NormFinder and geNorm software. We determined that genes encoding 18S rRNAs, actins and tubulins, currently used in tea plant studies were the most unstable reference genes, while specific genes *CsPTB1*, *CsEF1*, *CsSAND1*, *CsCLATHRIN1* and *CsUBC1* were the most appropriate reference genes for qRT-PCR analysis in tea plant under complex experimental conditions.

## 2. Results

To identify stable reference genes for gene expression studies in tea plant, 11 candidate reference genes and two target genes were investigated by qRT-PCR using six series of samples. First, the primer specificity was detected based on melting curve analysis and agarose gel electrophoresis analysis using pooled cDNAs as template. Results showed that at 53 °C annealing temperature, all primer pairs amplified a single major peak with all samples. Only *CsUBQ1* contained a second minor shoulder peak at 53 °C or lower temperatures (Supplemental Figure S1). After 45 cycles of amplification, the PCR products were examined on a 1.5% agarose gel, and all the tested amplicons produced a single visible band of the expected size (Supplemental Figure S2). We also checked the primer specificity performance at a wide range of annealing temperature from 50 to 59 °C with intervals of 3 °C. Melting curve analysis indicated that all the designed primers worked well at all of the tested annealing temperature, except for *CsUBQ1* which was not specific when amplified at an annealing temperature of 53 °C (Supplemental Table S1). The efficiency of PCR amplification, defined as percentage of PCR product increase per cycle, was determined for each gene using two-fold serial dilution of the pooled cDNAs (Table 1). Resulted showed that our primers which amplified *Cs18S* *rRNA1*, *CsACTIN1*, *CsCLATHRIN1*, *CsEF1*, *CsSAND1*, *CsTIP1* and *CsUBQ1* had excellent amplification efficiency of between 98% and 106%, while *CsGAPDH1*, *CsTUBULIN1*, *CsUBC1* and *CsPTB1* had less but still acceptable efficiencies of between 92% and 95%.

**Table 1:** Description of tea plant reference genes for qRT-PCR.

Reference Gene	GeneBank Accession Number	*Arabidopsis* Ortholog	*Arabidopsis* Locus Description	Forward/Reverse Primer Sequence (5'–3')	Amplicon Size (bp)	qRT-PCR Efficiency (%)
*Cs18S rRNA1*	AY563528.1	At3g41768	18S ribosomal RNA	TGGCCTTCGGGATCGGAGTAAT/GCTTTCGCAGTTGTTCGTCTTTCAT	108	101
*CsACTIN1*	KA280216.1	At5g09810	Actin 2/7	TGGGCCAGAAAGATGCTTATGTAGG/ATGCCAGATCTTTTCCATGTCATCC	118	103
*CsCLATHRIN1*	KA291473.1	AT5G46630	Clathrin adaptor complex subunit	TAGAGCGGGTAGTGGAGACCTCGTT/TACCAAAGCCGGCTCGTATGAGATT	129	102
*CsEF1*	KA280301.1	AT5G60390	Elongation factor 1 alpha	TTGGACAAGCTCAAGGCTGAACG/ATGGCCAGGAGCATCAATGACAGT	110	98
*CsGAPDH1*	KA295375.1	AT1G13440	Glyceraldehyde-3-phosphate dehydrogenase	TTTTTGGCCTTAGGAACCCAGAGG/GGGCAGCAGCCTTATCCTTATCAGT	107	93
*CsSAND1*	KM057790	AT2G28390	SAND family protein	TCCAATTGCCCCCTTAATGACTCA/GTAAGGGCAGGCAAACACCAGGTA	109	106
*CsTIP1*	KA283116.1	AT4G34270	TIP41-like protein	GGGTGCTTATGAGATTGAGGGACAC/ATATGCCGCAGAATCAGATGGGTAT	148	101
*CsTUBULIN1*	DQ444294.1	XM_002871860	Tubulin alpha-3	TGCCGCTAATAACTTTGCCAGAGG/GCCACCGCCAACAGCGTTAGTA	141	92
*CsUBQ1*	HM003234.1	AT4G05320	Ubiquitin	GCCGGAAAACAGCTTGAAGATGG/AGGACGGCTCAATAATCCCACCAC	111	98
*CsUBC1*	KA281185.1	AT4G27960	Ubiquitin-conjugating enzyme	TGCTGGTGGGGTTTTTCTTGTTACC/AAGGCATATGCTCCCATTGCTGTTT	124	92
*CsPTB1*	GAAC01052498.1	AT3G01150	Polypyrimidine tract-binding protein	TGACCAAGCACACTCCACACTATCG/TGCCCCCTTATCATCATCCACAA	107	95

### 2.1. Expression Profiles of Candidate Reference Genes

**Figure 1:**
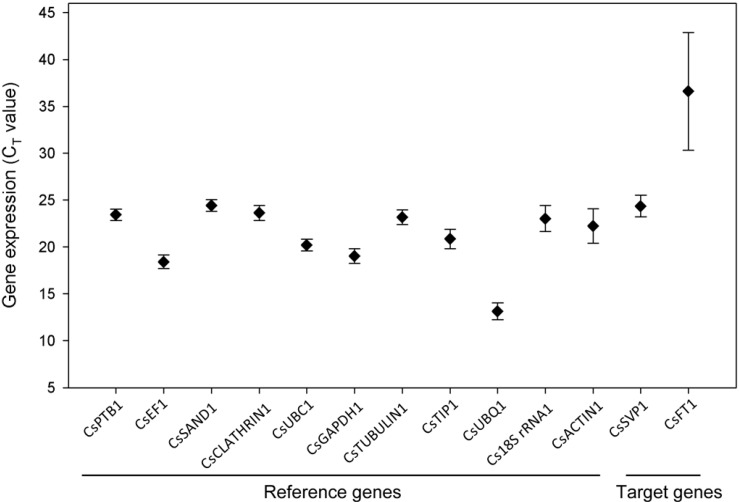
Average cycle threshold (*C*_T_) values for 11 candidate reference genes. The filled diamond symbol indicates the mean of *C*_T_ values. The bars indicate standard deviation.

Based on radioactive tracer normalized and quantified cDNA template, gene expression analysis of the 11 candidate reference gene exhibited a narrow *C*_T_ range among all the experimental series (Figure 1). The *C*_T_ value ranged from 13.1 to 24.4, while the majority of these values were distributed between 20 and 23. The transcript of *Cs18S rRNA1* was the most abundant (with lowest *C*_T_ value of 13.1) housekeeping gene, while the *CsSAND1* gene produced the least abundant transcript with a *C*_T_ value of 24.4. Each reference genes had varied expression ranges across all studied cDNA samples. *CsPTB1*, *CsSAND1* and *CsUBC1* had the lowest transcript variations among studied reference genes, while Cs*UBQ1*, *CsACTIN1* and *CsTUBULIN1* showed the highest expression variations. For target genes, *CsSVP1* gene had a mean *C*_T_ value of 24.4, while *CsFT1* had a very high *C*_T_ value of 36.6. *CsFT1* gene also had a more dynamic range of transcript accumulation compared to *SVP1* and the various reference genes. Most of the reference genes studied had smaller expression variation compared to the variation of target genes.

**Figure 2:**
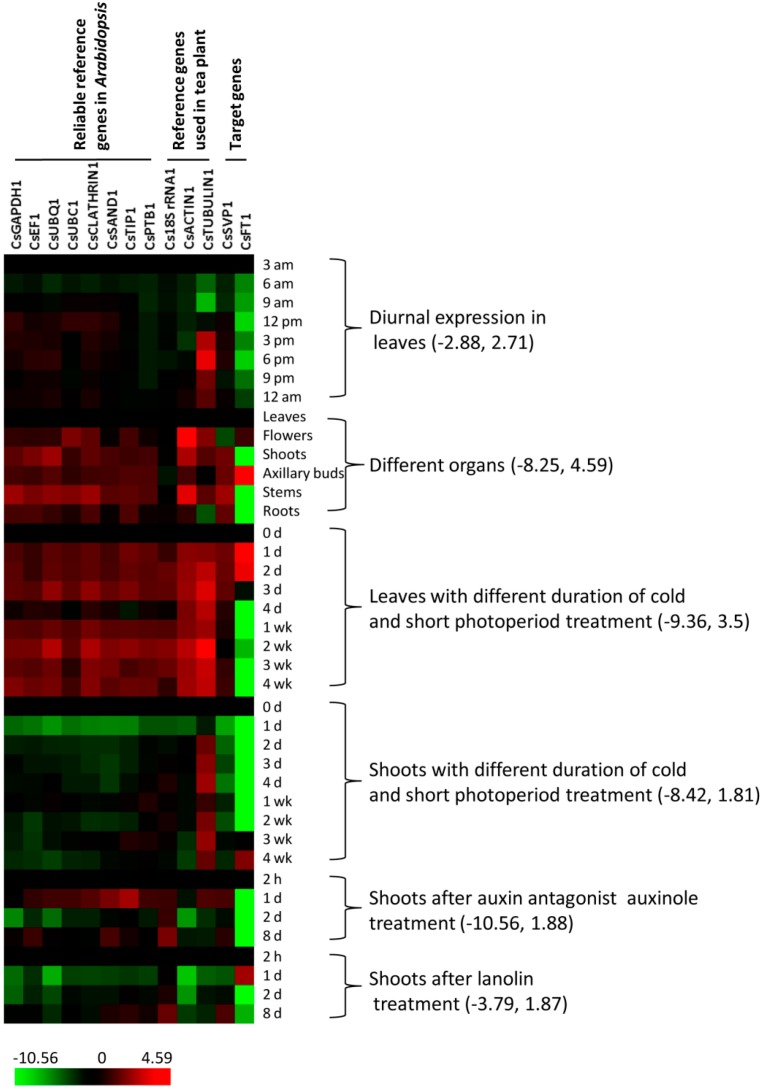
Expression profile of candidate reference genes and target genes under different experimental conditions. Six experimental series including 94 samples were used to examine gene expression. The fold difference is designated as log2 value. Red indicates up-regulated genes and green indicates down-regulated genes as compared with first sampling point/tissue. Bars at the bottom indicate the range of transcript changes in log2 value. The range of transcript changes for each experimental series is provided inside the parenthesis.

Expression profiles of candidate reference genes and target genes are shown in Figure 2. In each series of experiments, the first sampling time point or organ was chosen as the control. Differences in gene expression relative to the chosen control were presented as ratios of log2 transformed relative expression values. Results showed that the target gene of *CsFT1* had significant expression fluctuation among the six experimental series, while another target gene of *CsSVP1* had relatively stable expression. Except for *CsACTIN1* and *CsTUBULIN1*, most of the candidate reference genes had relatively more stable expression in different experimental series compared with the target genes. During the diurnal expression in leaves, *CsTUBULIN1*was significantly down-regulated at 9:00 am and up-regulated at 6:00 pm, while all of the other candidate reference genes had relatively stable expression. *CsACTIN1* and *CsTUBULIN1* had clear differential transcript abundance in different organs, but the expressions of *CsSAND1*, *CsTIP1* and *CsPTB1* were consistent across the different organs tested. *CsACTIN1* and *CsTUBULIN1* were highly induced in leaves after cold treatment. *CsUBQ1*, *CsCLATHRIN1* and *CsTIP1* also had significant expression variations at various time points during the cold treatment. In shoots, no significant expression fluctuation was observed with most reference genes at 1 day of cold and short photoperiod treatment, except for the substantial repression of *CsUBQ1*, *CsCLATHRIN1* and *CsTIP1*, and the observed increase in accumulation of the *CsTUBULIN1* transcript. Following auxinole (an auxin action inhibitor) and lanolin treatment, the expression of *CsACTIN1*, *CsGAPDH1*, *CsUBQ1*, *CsTIP1* and *Cs18S rRNA1* varied substantially in shoots. Among all the candidate reference genes, *CsACTIN1* and *CsTUBULIN1* were the most unstable, while *CsPTB1* seemed to be most stable in all of the experimental series.

### 2.2. Stability Ranking of Candidate Reference Genes

Four different computation programs geNorm, Normfinder, BestKeeper, and comparative ∆*C*_T_ were used to rank the candidate reference genes based on *C*_T_ values of 94 samples (Supplemental Table S2) from different experimental series. The stability ranking results are shown in Table 2. Additionally, the analytical tool RefFinder also gave a recommended comprehensive ranking for each gene based on weight assignment (Table 2, details were presented in Supplemental Figure S3). Based on this analysis, *CsEF1*, *CsGAPDH1*, *CsCLATHRIN1* and *CsSAND1* were the top four reference genes during the diurnal expression analysis. In contrast, *CsACTIN1* and *CsTUBULIN1* were the worst reference genes. In different organs, *CsCLATHRIN1*, *CsPTB1* and *CsSAND1* were most stable, while *CsUBQ1*, *CsACTIN1* and *CsTUBULIN1* were the most unstable genes. In leaves with cold and short photoperiod treatment, the three most stably expressed genes were *CsCLATHRIN1*, *CsGAPDH1* and *CsUBQ1* and the four least stable were *Cs18S rRNA1*, *CsACTIN1*, *CsTIP1* and *CsTUBULIN1*. Likewise, in shoots with cold and short photoperiod treatment, *CsUBC1*, *CsPTB1*, *CsGAPDH1* and *CsCLATHRIN1* were the best reference genes and had very similar scores. On the contrary, *CsACTIN1* and *CsTUBULIN1* were again the least stably expressed. After auxinole treatment, *CsPTB1*, *CsCLATHRIN1* and *CsUBC1* expression were most stable and *CsTIP1*, *CsUBQ1*, *CsGAPDH1*, *Cs18S rRNA1* and *CsACTIN1* were relatively less stably expressed. In shoots with lanolin treatment, *CsUBC1*, *CsPTB1*, *CsCLATHRIN1*, and *CsEF1* showed good expression stability, while *CsGAPDH1*, *Cs18S rRNA1*, *CsUBQ1* and *CsACTIN1* were not as stable. When all the sample data was included, the order of top ranked reference genes are *CsPTB1*, *CsEF1*, *CsSAND1*, *CsCLATHRIN1*, and *CsUBC1* and the order of worst ranked genes are *CsACTIN1*, *CsTUBULIN1*, *Cs18S rRNA1* and *CsUBQ1*.

## 3. Discussion

In this study, 11 candidate reference genes were selected from currently used reference genes in tea research, orthologs of *Arabidopsis* genes including superior traditional reference genes, and those identified as stably expressed in *Arabidopsis* whole-genome GeneChip studies [16,27]. The stability of these candidate reference genes were evaluated by qRT-PCR based on relative cDNA quantification, which is a reliable method to evaluate reference gene stability [28]. Results indicated that the primers had very good specificity through a wide range of annealing temperatures (Supplemental Figures S1 and S2, Table S1). In addition, qRT-PCR for each tested reference gene showed high efficiency (Table 1). These observations suggest our primer design for analyses of reference gene stability in this study is reliable, and, with the exception of the primers for *CsUBQ1*, were likely gene-specific.

**Table 2:** Stability ranking of candidate reference genes.

Analysis Tool	Ranking Order (1 being the most stable, 11 being the least stable)										
	1	2	3	4	5	6	7	8	9	10	11
*Diurnal Expression Ranking Order*											
**∆*C*_T_**	*CsEF1*	*CsGAPDH1*	*CsSAND1*	*CsCLATHRIN1*	*CsTIP1*	*CsUBQ1*	*CsUBC1*	*CsPTB1*	*Cs18S rRNA1*	*CsACTIN1*	*CsACTIN1*
**BestKeeper**	*Cs18S rRNA1*	*CsPTB1*	*CsEF1*	*CsSAND1*	*CsTIP1*	*CsGAPDH1*	*CsUBC1*	*CsCLATHRIN1*	*CsACTIN1*	*CsUBQ1*	*CsACTIN1*
**Normfinder**	*CsEF1*	*CsGAPDH1*	*CsUBQ1*	*CsCLATHRIN1*	*CsSAND1*	*CsTIP1*	*CsPTB1*	*CsUBC1*	*Cs18S rRNA1*	*CsACTIN1*	*CsACTIN1*
**Genorm**	*CsGAPDH1|CsCLATHRIN1*		*CsSAND1*	*CsEF1*	*CsTIP1*	*CsUBC1*	*CsUBQ1*	*CsPTB1*	*Cs18S rRNA1*	*CsACTIN1*	*CsACTIN1*
**Recommended comprehensive ranking**	*CsEF1*	*CsGAPDH1*	*CsCLATHRIN1*	*CsSAND1*	*Cs18S rRNA1*	*CsTIP1*	*CsPTB1*	*CsUBQ1*	*CsUBC1*	*CsACTIN1*	*CsACTIN1*
*Different Organs Ranking Order*											
**∆*C*_T_**	*CsCLATHRIN1*	*CsEF1*	*CsGAPDH1*	*CsPTB1*	*CsSAND1*	*CsTIP1*	*CsUBC1*	*CsUBQ1*	*Cs18S rRNA1*	*CsACTIN1*	*CsACTIN1*
**BestKeeper**	*Cs18S rRNA1*	*CsPTB1*	*CsSAND1*	*CsTIP1*	*CsCLATHRIN1*	*CsEF1*	*CsUBC1*	*CsGAPDH1*	*CsUBQ1*	*CsACTIN1*	*CsACTIN1*
**Normfinder**	*CsCLATHRIN1*	*CsUBC1*	*CsGAPDH1*	*CsEF1*	*CsTIP1*	*CsPTB1*	*CsSAND1*	*CsUBQ1*	*Cs18S rRNA1*	*CsACTIN1*	*CsACTIN1*
**Genorm**	*CsPTB1|CsSAND1*		*CsEF1*	*CsGAPDH1*	*CsCLATHRIN1*	*CsUBQ1*	*CsTIP1*	*CsUBC1*	*Cs18S rRNA1*	*CsACTIN1*	*CsACTIN1*
**Recommended comprehensive ranking**	*CsCLATHRIN1*	*CsPTB1*	*CsSAND1*	*CsEF1*	*CsGAPDH1*	*Cs18S rRNA1*	*CsUBC1*	*CsTIP1*	*CsUBQ1*	*CsACTIN1*	*CsACTIN1*
*Leaves with Cold and Short Photoperiod Treatment Ranking Order*											
**∆*C*_T_**	*CsCLATHRIN1*	*CsGAPDH1*	*CsPTB1*	*CsUBQ1*	*CsSAND1*	*CsUBC1*	*CsACTIN1*	*CsEF1*	*Cs18S rRNA1*	*CsTIP1*	*CsACTIN1*
**BestKeeper**	*CsEF1*	*CsSAND1*	*Cs18S rRNA1*	*CsGAPDH1*	*CsUBC1*	*CsPTB1*	*CsCLATHRIN1*	*CsACTIN1*	*CsTIP1*	*CsUBQ1*	*CsACTIN1*
**Normfinder**	*CsCLATHRIN1*	*CsGAPDH1*	*CsUBQ1*	*CsPTB1*	*CsSAND1*	*CsUBC1*	*CsACTIN1*	*CsEF1*	*Cs18S rRNA1*	*CsTIP1*	*CsACTIN1*
**Genorm**	*CsCLATHRIN1|CsUBQ1*		*CsGAPDH1*	*CsPTB1*	*CsUBC1*	*CsSAND1*	*CsEF1*	*CsACTIN1*	*Cs18S rRNA1*	*CsTIP1*	*CsACTIN1*
**Recommended comprehensive ranking**	*CsCLATHRIN1*	*CsGAPDH1*	*CsUBQ1*	*CsPTB1*	*CsSAND1*	*CsEF1*	*CsUBC1*	*Cs18S rRNA1*	*CsACTIN1*	*CsTIP1*	*CsACTIN1*
*Shoots with Cold and Short Photoperiod Treatment Ranking Order*											
**∆*C*_T_**	*CsUBC1*	*CsGAPDH1*	*CsPTB1*	*CsCLATHRIN1*	*CsUBQ1*	*CsEF1*	*CsSAND1*	*Cs18S rRNA1*	*CsTIP1*	*CsACTIN1*	*CsACTIN1*
**BestKeeper**	*CsPTB1*	*CsGAPDH1*	*CsUBC1*	*CsCLATHRIN1*	*CsEF1*	*Cs18S rRNA1*	*CsACTIN1*	*CsSAND1*	*CsTIP1*	*CsUBQ1*	*CsACTIN1*
**Normfinder**	*CsUBC1*	*CsGAPDH1*	*CsPTB1*	*CsCLATHRIN1*	*CsUBQ1*	*CsEF1*	*Cs18S rRNA1*	*CsSAND1*	*CsTIP1*	*CsACTIN1*	*CsACTIN1*
**Genorm**	*CsUBC1|CsCLATHRIN1*		*CsPTB1*	*CsTIP1*	*CsSAND1*	*CsGAPDH1*	*CsUBQ1*	*CsEF1*	*Cs18S rRNA1*	*CsACTIN1*	*CsACTIN1*
**Recommended comprehensive ranking**	*CsUBC1*	*CsPTB1*	*CsGAPDH1*	*CsCLATHRIN1*	*CsEF1*	*CsUBQ1*	*CsSAND1*	*CsTIP1*	*Cs18S rRNA1*	*CsACTIN1*	*CsACTIN1*
*Auxin Antagonist Auxinole Treatment Ranking Order*											
**∆*C*_T_**	*CsPTB1*	*CsCLATHRIN1*	*CsUBC1*	*CsSAND1*	*CsEF1*	*CsACTIN1*	*CsUBQ1*	*CsTIP1*	*CsGAPDH1*	*CsACTIN1*	*Cs18S rRNA1*
**BestKeeper**	*CsPTB1*	*CsUBC1*	*CsCLATHRIN1*	*CsACTIN1*	*CsEF1*	*Cs18S rRNA1*	*CsSAND1*	*CsACTIN1*	*CsGAPDH1*	*CsTIP1*	*CsUBQ1*
**Normfinder**	*CsPTB1*	*CsCLATHRIN1*	*CsUBC1*	*CsSAND1*	*CsEF1*	*CsACTIN1*	*CsUBQ1*	*CsTIP1*	*CsGAPDH1*	*CsACTIN1*	*Cs18S rRNA1*
**Genorm**	*CsPTB1|CsCLATHRIN1*		*CsUBC1*	*CsACTIN1*	*CsSAND1*	*CsTIP1*	*CsEF1*	*CsUBQ1*	*CsGAPDH1*	*CsACTIN1*	*Cs18S rRNA1*
**Recommended comprehensive ranking**	*CsPTB1*	*CsCLATHRIN1*	*CsUBC1*	*CsSAND1*	*CsACTIN1*	*CsEF1*	*CsTIP1*	*CsUBQ1*	*CsGAPDH1*	*Cs18S rRNA1*	*CsACTIN1*
*Lanolin Treatment Ranking Order*											
**∆*C*_T_**	*CsPTB1*	*CsUBC1*	*CsCLATHRIN1*	*CsEF1*	*CsSAND1*	*CsTIP1*	*CsACTIN1*	*CsGAPDH1*	*Cs18S rRNA1*	*CsUBQ1*	*CsACTIN1*
**BestKeeper**	*CsEF1*	*CsUBC1*	*CsPTB1*	*CsCLATHRIN1*	*CsSAND1*	*CsTIP1*	*CsACTIN1*	*Cs18S rRNA1*	*CsGAPDH1*	*CsUBQ1*	*CsACTIN1*
**Normfinder**	*CsPTB1*	*CsUBC1*	*CsCLATHRIN1*	*CsSAND1*	*CsEF1*	*CsTIP1*	*CsACTIN1*	*CsGAPDH1*	*Cs18S rRNA1*	*CsUBQ1*	*CsACTIN1*
**Genorm**	*CsUBC1|CsCLATHRIN1*		*CsEF1*	*CsPTB1*	*CsTIP1*	*CsSAND1*	*CsACTIN1*	*CsGAPDH1*	*Cs18S rRNA1*	*CsUBQ1*	*CsACTIN1*
**Recommended comprehensive ranking**	*CsUBC1*	*CsPTB1*	*CsCLATHRIN1*	*CsEF1*	*CsSAND1*	*CsTIP1*	*CsACTIN1*	*CsGAPDH1*	*Cs18S rRNA1*	*CsUBQ1*	*CsACTIN1*
*All Samples Ranking Order*											
**∆*C*_T_**	*CsEF1*	*CsCLATHRIN1*	*CsGAPDH1*	*CsPTB1*	*CsUBC1*	*CsSAND1*	*CsTIP1*	*CsUBQ1*	*Cs18S rRNA1*	*CsACTIN1*	*CsACTIN1*
**BestKeeper**	*CsPTB1*	*CsSAND1*	*CsUBC1*	*CsTIP1*	*CsEF1*	*CsGAPDH1*	*CsCLATHRIN1*	*Cs18S rRNA1*	*CsUBQ1*	*CsACTIN1*	*CsACTIN1*
**Normfinder**	*CsCLATHRIN1*	*CsEF1*	*CsGAPDH1*	*CsUBC1*	*CsPTB1*	*CsUBQ1*	*CsTIP1*	*CsSAND1*	*Cs18S rRNA1*	*CsACTIN1*	*CsACTIN1*
**Genorm**	*CsPTB1|CsSAND1*		*CsUBC1*	*CsTIP1*	*CsEF1*	*CsGAPDH1*	*CsCLATHRIN1*	*CsUBQ1*	*Cs18S rRNA1*	*CsACTIN1*	*CsACTIN1*
**Recommended comprehensive ranking**	*CsPTB1*	*CsEF1*	*CsSAND1*	*CsCLATHRIN1*	*CsUBC1*	*CsGAPDH1*	*CsTIP1*	*CsUBQ1*	*Cs18S rRNA1*	*CsACTIN1*	*CsACTIN1*

The stability of candidate reference genes was validated under different experimental series, including diurnal expression in leaves, different organs, leaves and shoots with cold and short photoperiod treatment, auxinole and lanolin treatment. The experimental conditions were chosen based on the current focuses in physiological and molecular studies of tea plant. The expressions of two target genes *CsSVP1* and *CsFT1* were examined as negative controls since they were expected to show variation in expression in most of the sample treatments tested. In *Arabidopsis*, SVP plays an important role in the response of plants to ambient temperature changes [29]. FT, as a member of the small phosphatidylethanolamine-binding protein family, is known to have a tissue-specific gene expression and can be affected by light, photoperiod, temperature and hormone [30]. As expected, the transcript accumulation profile of *CsSVP1* was significantly suppressed in shoots after cold and short photoperiod treatment. Likewise, *CsFT1* gene had significant and expected expression fluctuation across the entire experimental series (Figure 2). The observed expected expression patterns of the target genes suggests that the relative quantitation of the cDNAs were accurate and that there were no obvious differences resulting from altered amplification efficiencies between samples in any given experiment. Thus, the stability rankings of 11 candidate reference genes is also likely reliable. Due to the differences in statistical algorithms, the four computational programs did not place the order of top ranked genes equally. In each experimental series, a recommend comprehensive ranking was given based on the ranking results from the four analysis programs. Unsurprisingly, no single gene showed stable transcript accumulation across all treatments tested. Consequently, this suggests that reference gene choice should be tested for any given experimental design to insure accurate quantitation of target RNAs. *CsUBC1* was the most stable reference gene in both shoots with cold and short photoperiod treatment and lanolin treatment series but performed poorly in diurnal expression series. Above results further indicated that it is difficult to identify a single universally applicable reference gene covering all conditions.

Although no single gene showed consistent expression transcript accumulation over all treatments, several reference genes performed better than the others, suggesting that these genes might be a good choice for reference genes under different experimental settings in tea plants. According to the resulting overall ranking, *CsPTB1*, *CsEF1*, *CsSAND1*, *CsCLATHRIN1* and *CsUBC1* were the top five ideal reference genes (Table 2). *CsPTB1* was chosen as a candidate reference gene because it was stably expressed in *Arabidopsis* across multiple Affymetrix ATH1 whole-genome GeneChip studies [27]. This gene was also subsequently identified as a stably expressed gene in leafy spurge suggesting it is a good first choice in other plant species [8]. *CsPTB1* gene encodes polyprimidine tract-binding protein which is involved in the regulation of pre-mRNA alternative splicing, internal ribosomal entry site-mediated translation and mRNA localizing/sorting [31]. Though the *CsPTB1* gene only ranked first at auxinole treatment series, it was ranked among the four top genes during all the experimental series except diurnal expression. Therefore, we suggest *CsPTB1* to be the best reference gene for tea plant in experiments not involving diurnal expression changes. *CsSAND1*, *CsCLATHRIN1*, and *CsEF1* genes were the next in ranking for top reference genes. *CsSAND1* was first identified in animal studies and had been validated as a good reference gene in *Arabidopsis*, leafy spurge, citrus, and grape [8,32,33,34,35,36]. Clathrins are important scaffolding proteins in plant endocytosis and also showed stable character in other reference gene selection studies [37,38,39,40]. *CsEF1*, which encodes a protein that plays an important role in translation and cytoskeletal rearrangements [41], was perhaps the most superior traditional reference gene [27], and similar genes demonstrated stable expression in different tissue of *Brassica rapa* [42], during developmental process in *Populus* [43], and under the effects of different biotic factors (development stage, tissue, and strain) in *Plutella xylostella* [44]. In our study, *CsEF1* gene was the most stable reference gene in diurnal expression series and also had good ranking in other experimental serials.

*CsACTIN1*, *CsTUBULIN1* and *Cs18S rRNA1* performed poorly among the 11 selected candidate genes in the experimental series of this study. This is likely due to the fact that these genes are members of larger gene families, and it has been demonstrated that individual members of these genes families have tissue specific and/or differential expression in response to environmental cues [45,46,47]. Thus, our results regarding the unstable expression of individual members of these gene families is not surprising and likely not unique to studies in tea plant. Indeed, the poor stability of *CsACTIN1*, *CsTUBULIN1* and *Cs18S rRNA1*, which were considered as ideal internal controls in qRT-PCR previously, have been repeatedly reported recently [25,26,48,49]. Wong and Medrano [6] pointed out that the stability of these genes has to be validated under one’s own experimental condition before using them as reference genes. Several previous studies have used *CsACTIN1* and *Cs18S rRNA1* genes in tea plant as controls to examine various expression patterns of other genes [19,24]. Even compared with the target gene *CsSVP1*, the expression variation of *CsACTIN1* and *CsTUBULIN1* was greater in most of the experimental treatments. Therefore, these genes are not reliable reference genes for gene expression research in tea or most other systems. Our observation suggests that previous studies which used these common tea plant reference genes may need to re-examine their work in light of our findings, and our results should provide a guideline for future reference gene choice for gene expression studies in tea plant.

## 4. Experimental Section

### 4.1. Plant Materials and Treatments

Three-year-old potted small leaf tea plant (*Camellia sinensis* (L.) O. Kuntze) was grown in a greenhouse under a 16 h photoperiod at 23–25 °C for one year. Regular fertilization and pest management were applied. The fourth to sixth intact mature leaves away from the terminal growing point were sampled at different time of a day (3:00 am, 6:00 am, 9:00 am, 12:00 pm, 3:00 pm, 6:00 pm 9:00 pm and 12:00 am) for validation of reference genes during diurnal cycles. Different organs, including mature leaves (the fourth to sixth intact leaves away from the terminal growing point), whole flowers at half boom, shoots (including a bud and a leaf), five-month-old stems (generated in spring), axillary buds (taken from one-year-old branches) and roots, were collected separately at 9:00 pm. For analysis of transcript accumulation during cold stress, greenhouse-grown tea plants were moved to a growth chamber set for a 10 h photoperiod at 10 °C. Both mature leaves (the fourth to sixth intact leaves away from the terminal growing point) and shoots (including a bud and a leaf) were collected after 0 day, 1 day, 2 day, 3 day, 4 day, 1 week, 2 week, 3 week and 4 week cold treatment. In addition, an auxin antagonist (auxinole, kindly provided by Ken-ichiro Hayashi, Okayama University of Science, Okayama, Japan [50,51]) was applied to the growing apical buds of greenhouse-grown tea plants as a 2 mM solution in lanolin. Auxinole preparation and treatment were followed the description by Iino [52]. Same number of apical buds was treated with lanolin as control treatment. Shoots were harvested after 2 h, 1 day, 2 day, and 8 day auxinole and lanolin treatment. Two to three biological replicates were collected from each treatment. All the samples were immediately frozen in liquid nitrogen and stored at −80 °C until extraction of RNA.

### 4.2. Total RNA Extraction and cDNA Template Preparation

Total RNA extractions were performed following the pine tree extraction method [53]. The extracted RNAs were quantified using a NanoDrop^®^ ND-100 Spectrophotometer (Thermo Fisher Scientific, Waltham, MS, USA) and the quantity and quality of RNAs was verified by 1% gel electrophoresis. For each sample, 5 µg RNA was used for cDNA synthesis following DNase treatment which was carried out using DNase I kit (Invitrogen, Carlsbad, CA, USA). cDNA synthesis was carried out using SuperScript^®^ III First-Strand Synthesis System (Invitrogen, Carlsbad, CA, USA). 0.4 µCi of [α-^32^P]-dCTP (PerkinElmer, Waltham, MS, USA) was added with dNTP mix during cDNA synthesis. To filter out free [α-^32^P]-dCTP, the final cDNA product was purified using a QIAquick^®^ PCR Purification Kit (QIAGEN, Valencia, CA, USA) and eluted with 50 µL final dilution of water. Quantitation of the resulting cDNA was determined by liquid scintillation as follows: 2 µL of eluted cDNA was mixed with 10 mL of scintillation cocktail (MP BIOMEDICALS, Irvine, CA, USA) and incorporated radioactivity was detected on Beckman LS 6000 Scintillation Counter (GMI, Albertville, MN, USA). The quantity of all the cDNA products was normalized based on the incorporated radioactivity of the least concentrated sample and then all samples were diluted 20-fold with ultrapure water. The final cDNA templates were stored in −80 °C for qRT-PCR analysis.

### 4.3. Primer Design and Quantitative Real-Time PCR (qRT-PCR)

All candidate reference genes were identified by BLASTX of the *Arabidopsis* reference genes against a tea plant EST database developed from assembly of the combined data from the small read archive (SRA) for tea plant at NCBI (http://www.ncbi.nlm.nih.gov/) (accession numbers: SRX020193, SRX147828, SRX153144, SRX202319, SRX149415). The sequences with high sequence similarity to each candidate reference genes were aligned separately to highlight potential areas of polymorphism. The primer pairs were designed by PrimerSelect of Lasergene 8 (DNASTAR, Inc., Madison, WI, USA) following the recommendations by Udvardi *et al.* [54], and primer pairs were selected from sequence region with the fewest polymorphisms. The primer pair information is provided in Table 1. The amplification specificity of designed primers was confirmed via standard qRT-PCR procedures under different annealing temperature (50, 53, 56 and 59 °C). PCR amplification efficiency was calculated using a two-fold serial dilution of the cocktail of cDNA templates following the description by Chao *et al.* [8]. All PCR products were examined by melting curve analysis and agarose gel electrophoresis.

Gene expression was examined by qRT-PCR on a LightCycle^®^ 480 Real-Time PCR System (Roche, Indianapolis, IN, USA). For real-time PCR reaction, 4 µL cDNA template was added to a 10 µL PCR reaction mixture containing 5 µL of 2× LightCycle^®^ 480 SYBR Green I Master (Roche, Indianapolis, IN, USA) and 0.5 µL of each primer (20 pmol). Thermal cycling was performed with the program setting of 10 min pre-incubation at 95 °C, 45 cycles amplification of 20 s at 95 °C, 10 s at appropriate annealing temperatures (56 °C for *CsACTIN1*, *Cs18S rRNA1*, *CsEF1*, *CsGAPDH1*, *CsTUBULIN1*, *CsUBC1* and *CsPTB1* and 59 °C for *CsUBQ1*, *CsCLATHRIN1*, *CsSAND1* and *CsTIP1*), and 35 s at 70 °C, and followed by dissociation analysis with a temperature ramp step added to the end of the amplification with an initial temperature of 65 °C and a final temperature of 97 °C. Three technical replicates were carried out for each reaction.

To validate the normalization effect of cDNA templates and the stability of candidate reference genes, the expressions of two target genes *CsSVP1* (Accession number: KM057787, forward primer: 5'-ACATTACGGCGAGGCAAGTGAC-3', reverse primer: 5'-CGGTGGCGGAGAAGATGATG-3') and *CsFT1* (Accession number: KC149523, forward primer: 5'-CCTTTACAAGGTCTATCTCTCTCAGG-3', 5'-TATATGATGATGATGATGTGA-3'), were included as negative controls since it was expected that these two genes would likely have differential transcript accumulation in many of the experimental treatments we were testing. We tested the expressions of these target genes under the same qRT-PCR conditions except with 54 and 60 °C annealing temperature, respectively. The average mean and the fluctuation range of the cycle threshold (*C*_T_) values between candidate reference genes and target genes were compared. Heat-maps of gene expression profile were performed following the description by Chao *et al.* [8].

The stabilities of candidate reference genes were determined based on a web-based analysis tool RefFinder (http://www.leonxie.com/referencegene.php). RefFinder is a comprehensive tool which consists of four computational programs geNorm, Normfinder, BestKeeper, and the comparative ∆*C*_T_ method. The expression stability of each tested candidate reference gene was compared and ranked by each program on the basis of their cycle threshold (*C*_T_) values. Furthermore, based on the rankings from each program, an individual gene was assigned an appropriate weight and finally given an overall final ranking by calculating the geometric mean of their weights.

## 5. Conclusions

We evaluated 11 candidate reference genes under different experimental conditions using reliable qRT-PCR assay based on accurate cDNA normalization. Using the web-based analytical tool RefFinder, *CsPTB1*, *CsEF1*, *CsSAND1*, *CsCLATHRIN1* and *CsUBC1* appeared to be the top five most stable reference genes for all the sample pools. However, variability in even these top performing reference genes suggests that reference gene choice needs to be evaluated for each experimental condition. In addition, *CsUBQ1*, *Cs18S rRNA1*, *CsACTIN1* and *CsTUBULIN1* seemed to be unsuitable as internal control among most experimental conditions we tested. Our study highlights problems with the currently used reference gene in tea plant for expression analysis under different conditions. Our results should prove useful for future works on expression analysis in tea plant using qRT-PCR.

## Supplementary Materials

Supplementary materials can be found at http://www.mdpi.com/1422-0067/15/12/22155/s1.
